# QIAstat-Dx Syndromic Molecular Testing Versus Conventional Diagnostics in Acute Gastroenteritis: Impact on Pathogen Detection and Laboratory Workflow

**DOI:** 10.3390/microorganisms14061345

**Published:** 2026-06-16

**Authors:** Fabio Formenti, Andrea Matucci, Martina Parisato, Marta Piccoli, Silvia Pasquetto, Milena Bernardi, Marco Venturini, Elena Pomari, Matteo Valerio, Cristina Mazzi, Marco Cavallini, Rebecca Passarelli Mantovani, Davide Treggiari, Chiara Piubelli, Francesca Perandin

**Affiliations:** 1 Department of Infectious, Tropical Diseases and Microbiology, IRCCS Sacro Cuore Don Calabria Hospital, Negrar di Valpolicella, 37024 Verona, Italy; 2 Medical Oncology Unit, IRCCS Sacro Cuore Don Calabria Hospital, Negrar di Valpolicella, 37024 Verona, Italy

**Keywords:** QIAstat, syndromic molecular diagnostics, acute gastroenteritis, multiplex PCR, stool culture, antigen detection

## Abstract

Acute gastroenteritis is a common condition with a wide and often indistinguishable etiology, requiring laboratory support for an accurate diagnosis. Classical diagnostic methods, including stool culture and antigen-based tests, are limited by restricted pathogen coverage and variable sensitivity. In the present study, 548 stool samples from patients with suspected gastroenteritis were tested using the QIAstat-Dx Gastrointestinal Panel 2 and compared with stool culture and rotavirus/adenovirus antigen tests. The molecular panel showed a positivity rate of 50.4%, consistently higher than stool culture (12.6%) and antigen assays (12.3% for rotavirus and 4.4% for adenovirus). The most frequently detected pathogens included enteropathogenic *Escherichia coli* (15.3%), *Campylobacter* spp. (12.0%), and enteroaggregative *E. coli* (10.2%). Agreement between methods was good for bacterial pathogens but low for viral targets. Discordant viral results were often associated with low antigen cut-off index values and multiple pathogen detections by the molecular panel, suggesting potential limitations of antigen-based assays. Overall, the QIAstat-Dx Gastrointestinal Panel 2 improves pathogen detection and provides rapid, comprehensive diagnostic information, while a combined approach with conventional methods may represent the most appropriate strategy for optimizing patient management.

## 1. Introduction

Acute gastroenteritis is a major cause of morbidity worldwide, affecting people of all ages and placing a consistent burden on healthcare systems and the economy [1,2]. Despite its typically self-limiting course, accurate and timely diagnosis is crucial for infection control purposes and to guide appropriate clinical management [3,4]. The spectrum of causative pathogens is wide and includes bacteria, viruses, and parasites [5], often presenting with overlapping clinical features that make etiological diagnosis based solely on symptoms [3].

The conventional diagnostic workflow for gastroenteritis is based on stool culture for bacterial pathogen identification and antigen-based tests for common enteric viruses, such as rotavirus and adenovirus [1,6]. Conventional diagnostic workflows also include microscopic examination of stool samples for the detection of parasitic infections [7]. Microscopic examination of stool samples is also associated with limited sensitivity, operator dependency, and the need for multiple specimens, which may reduce diagnostic yield [7]. While these methods remain widely used, they present several limitations: (i) stool culture is labor-intensive, (ii) requires trained personnel and (iii) is inherently time-consuming, often requiring 48–72 h or longer to obtain cultural results [1,8]. Moreover, its sensitivity can be suboptimal, particularly for fastidious or non-viable pathogens [9]. Indeed, some bacterial pathogens may enter a viable but non-culturable (VBNC) state, resulting in false-negative culture results despite the presence of viable organisms [10]. Furthermore, antigen detection tests can typically detect a limited number of viruses and may exhibit variable sensitivity/specificity depending on the assay used and the viral load in the sample [2]. Therefore, a substantial proportion of gastroenteritis cases remain undiagnosed when conventional diagnostic approaches are adopted [11].

In recent years, syndromic molecular panels have emerged as a promising alternative for the diagnosis of infectious diseases, including gastrointestinal infections [12,13,14,15,16,17]. These multiplex real-time PCR-based assays, such as the QIAstat-Dx gastrointestinal 2 panel (GI2), enable the simultaneous detection of a wide range of bacterial, viral, and parasitic pathogens directly from stool samples with significantly reduced turnaround times [6,8,16,18,19]. Several studies have demonstrated that these platforms can significantly increase pathogen detection rates compared to conventional methods, and they can identify coinfections that would otherwise go undiagnosed [5,6,7,11,20,21,22,23].

On the other hand, the increased analytical sensitivity of molecular assays raises important questions regarding the clinical significance of the detected pathogens, particularly in cases of low pathogen load or prolonged shedding [24]. Moreover, syndromic panels do not provide antimicrobial susceptibility data, which are essential for the management of certain bacterial infections and epidemiological surveillance [3,13]. Cost considerations and laboratory workflow implications also play a critical role in determining whether these technologies can be broadly implemented as first-line diagnostic tools [25].

In this context, the question extends beyond whether syndromic panels outperform conventional methods in diagnostic accuracy, focusing instead on whether they can meaningfully replace traditional approaches in routine clinical practice. To address this issue, it is necessary to consider not only diagnostic performance but also the impact on pathogen detection rates, the clinical relevance of findings, and implications for patient management and laboratory workflow organization [26,27].

The aim of the present study was to evaluate whether implementation of the QIAstat-Dx Gastrointestinal Panel 2 could represent a viable alternative to conventional diagnostic methods, including stool culture and rotavirus/adenovirus antigen tests, in a cohort of 548 patients with suspected acute gastroenteritis. By directly comparing these diagnostic approaches, we sought to assess differences in pathogen detection rates, agreement between methods, and their potential role in optimizing the laboratory diagnosis of gastroenteritis.

## 2. Materials and Methods

### 2.1. Study Design and Population

This diagnostic accuracy study aimed to assess the performance of a syndromic molecular assay for the diagnosis of acute gastroenteritis.

A total of 548 patients presenting with symptoms suggestive of gastrointestinal infection at IRCCS Sacro Cuore Don Calabria Hospital were included in the study. Stool samples were collected as part of routine clinical practice at the Laboratory of Microbiology, Department of Infectious Tropical Diseases and Microbiology, between December 2024 and November 2025. Subjects involved in the study or their legal representatives provided written informed consent.

Inclusion criteria included patients presenting with gastrointestinal symptoms. No exclusion criteria were applied regarding age or sex.

### 2.2. Microbiological Methods

#### 2.2.1. Molecular Methods

All samples collected in Cary-Blair transport medium (FecalSwab™, Copan Diagnostics, Brescia, Italy) were tested using the QIAstat-Dx GI2 panel cartridge on QIAstat-DX Analyzer (Qiagen, Hilden, Germany) according to the manufacturer’s instructions. Briefly, 200 µL of sample collected in Cary–Blair eSwab medium was dispensed into the QIAstat-DX cartridge. The QIAstat-Dx GI2 panel is a fully integrated multiplex real-time PCR assay that combines nucleic acid extraction, amplification, detection, and result interpretation within a single-use cartridge.

This multiplex real-time PCR allows the simultaneous detection of 17 targets (four viruses, eight bacteria plus the detection of *E. coli* O157 serogroup within STEC, and four parasite species). The panel targets are: adenovirus F40/F41, astrovirus, norovirus GI/GII, rotavirus A, *Campylobacter* (*C. jejuni*, *C. coli*, and *C. upsaliensis*), *Shigella*/Enteroinvasive *E. coli* (EIEC), Enteropathogenic *E. coli* (EPEC), Enterotoxigenic *E. coli* (ETEC), *P. shigelloides*, *Salmonella* spp., Shiga-like toxin-producing *E. coli* (STEC) stx1/stx2 including specific identification of *E. coli* O157 serogroup within STEC, *Y. enterocolitica*, *Cryptosporidium*, *C. cayetanensis*, *E. histolytica*, and *G. lamblia*. Sample preparation required approximately 2 min of hands-on time, while the total assay run time was approximately 70 min according to the manufacturer’s specifications Cartridge loading and multiplex PCR tests were conducted as per the manufacturer’s instructions. The cycle threshold (Ct) value of target amplification was recorded.

#### 2.2.2. Conventional Diagnostic Methods

All samples were tested using conventional diagnostic methods, including:

(A) Bacterial culture, performed on FecalSwab^TM^ samples, carried out using standard microbiological procedures for the detection of common enteric bacterial pathogens (e.g., *Salmonella* spp., *Shigella* spp. and *Campylobacter* spp.). Briefly, 10 µL of samples collected in Cary–Blair medium were inoculated using the WASP automated system onto the following media: chromID^TM^ Salmonella agar, Aeromonas agar, Hektoen enteric agar, and Karmali selective medium, as well as into Selenite F enrichment broth.
(B) Qualitative antigen detection, performed on fresh stool samples, of rotavirus and adenovirus, carried out using commercial fluorescent immunoassay slides (STANDARD F Rota/Adeno Ag FIA, SD Biosensor, Suwon, Republic of Korea) loaded on a STANDARD F2400 Analyzer (SD Biosensor, Suwon, Republic of Korea) according to the manufacturer’s instructions. The cut-off index (COI) of the positive samples was also collected.

### 2.3. Data Collection

For each patient, demographic and clinical data were collected when available, including age and sex. Results from both the molecular assay and conventional methods were recorded for each stool sample.

Discordant results between methods were further evaluated and categorized.

### 2.4. Statistical Analysis

Performance analysis was carried out using 2 × 2 contingency tables in GraphPad Prism version 11.0.0, considering conventional methods as the reference standard. Of the 548 collected samples, 9 were excluded because stool culture had not been performed in parallel, leaving 539 samples available for comparison between the GI2 panel and stool culture. All the 9 samples were GI2 panel-negative for *Salmonella* spp. and *Shigella* targets. For rotavirus and adenovirus, 405 samples were available for comparative analysis. A total of 143 samples were excluded because fresh stool specimens required for antigen testing were unavailable, leaving only FecalSwab^TM^ samples suitable for GI2 testing. Among these excluded samples, 1 was positive for Rotavirus A and 4 were positive for Adenovirus F40/F41 by GI2, while the remaining samples were negative for the respective targets. The positivity rate was calculated as the number of samples positive for at least one target divided by the total number of tested samples and expressed as a percentage.

Variables were summarized using descriptive statistics, including number of observations, mean, standard deviation, minimum, median, and maximum values. Categorical values were summarized using the number of observations and percentages. R statistical software (version 4.5.1) was used for analysis.

### 2.5. Ethical Considerations

This study was conducted in accordance with the Declaration of Helsinki and approved, using Protocol n° 53742, by the Ethical Committee of Verona and Rovigo provinces on 25 September 2024.

## 3. Results

### 3.1. Sample Distribution

A total of 548 patients presenting at IRCCS Sacro Cuore Don Calabria Hospital with suspected acute gastroenteritis were tested. Most of the diagnostic requests were from the emergency department (55.4%), followed by pediatrics (17.9%), general medicine (5.9%), gastroenterology (5.3%) and infectious diseases (3.5%), while 12% were submitted by other clinical units. Sex distribution within the study population was balanced, with 52.6% (288) of patients being male and 47.4% (260) female. Age distribution analysis revealed a higher frequency of test requests in younger patients, particularly in the 0–10 years (21.7%) and 10–20 years (18.6%) age groups. Lower proportions were observed in the intermediate age range of 20 to 70 years, while a further increase was noted in older patients, with 10.0% of tests in the 70–80 years group and 9.3% in those aged 80–90 years (Supplementary Table S1). During the study period, from December 2024 to November 2025, an average of approximately 50 samples (49.6 samples as geometric mean) per month (min-max; 30–67) were tested (Supplementary Figure S1).

### 3.2. Diagnostic Results with QIAstat-Dx GI2 Panel

**Figure 1:**
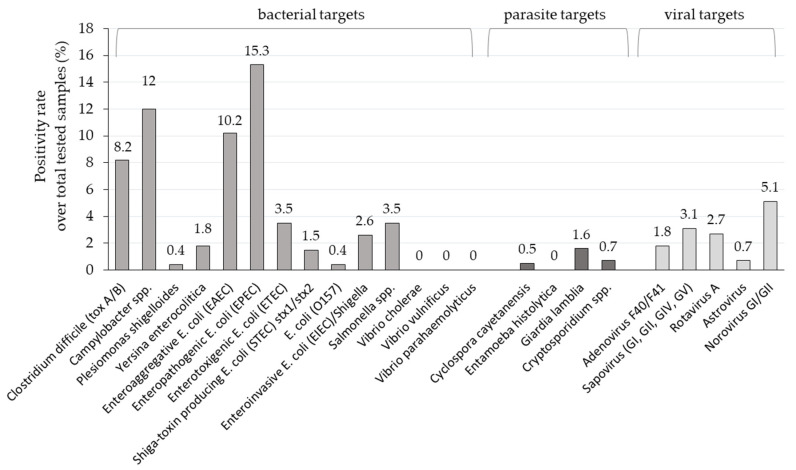
Percentage of detected pathogens over tested samples (N = 548).

The QIAstat-Dx GI2 panel showed an overall positivity rate of 50.4% for at least one target, with EPEC, EAEC and *Campylobacter* spp. being the most frequently detected targets (Figure 1). Single pathogen positivity was observed in 31.2% of samples, while 11.5% showed two pathogens. Higher orders of co-detections were less frequent but still observable up to six pathogens in a single sample (Supplementary Table S2). Positive target detection output data, represented by cycle threshold (Ct) values, varied across detected pathogens (Table 1).

### 3.3. Diagnostic Results with Conventional Methods

**Figure 2:**
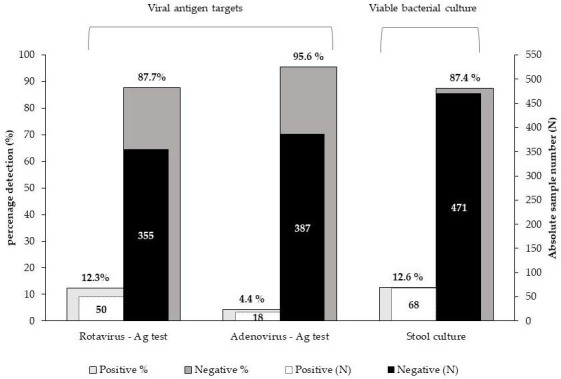
Percentage and absolute number of positive and negative results for viral antigen detection (rotavirus and adenovirus) using FIA tests and stool cultures for bacterial growth. Values refer to 405 samples for viral antigen tests and 539 samples for stool culture. Columns represent percentages, while inner columns represent the absolute number of negative and positive samples respectively.

Stool culture was positive for *Salmonella* spp. in 18/539 samples, and for *Campylobacter* spp. in 50/539 samples. No *Shigella* spp. growth was observed. Conventional cultural diagnostic methods detected bacterial growth in 12.6% of stool samples (68/539). Viral antigen tests showed positivity rates of 12.3% for rotavirus, with 50 antigen-positive samples out of 405 tested, and 4.4% for adenovirus, with 18 FIA positive samples out of 405 tested (Figure 2).

### 3.4. Comparison Between the Two Diagnostic Approaches

Agreement and performance between the syndromic panel and conventional diagnostic methods were assessed for shared targets. For bacterial pathogens, a high level of concordance was observed. Agreement between the molecular panel and stool culture for *Salmonella* spp. and *Campylobacter* spp. was almost perfect, with a Cohen’s kappa (κ) of 0.972 (95% CI: 0.917–1.000) and 0.863 (95% CI: 0.793–0.933) respectively (Table 2 and Supplementary Table S3). No *Shigella* spp. isolates were detected by stool culture, and therefore agreement could not be assessed. Moreover, relying solely on the conventional diagnostic methods routine to our laboratory would have resulted in missing all the enteropathogens identified by the molecular syndromic panel. In contrast, agreement between the syndromic panel and antigen tests was considerably lower. For rotavirus, Cohen’s kappa was 0.273 (95% CI: 0.119–0.427), while for adenovirus it was 0.351 (95% CI: 0.095–0.607), indicating poor concordance between the two approaches (Table 2). A substantial number of discordant results were observed, particularly for viral antigen detection tests. A total of 40 discrepant cases were identified between rotavirus antigen-positive samples testing negative on the GI2 panel, and 13 discrepancies were observed for adenovirus antigen-positive samples testing negative on GI2. Conversely, we observed 4 samples positive for rotavirus only on GI2 and 1 positive for adenovirus only on GI2. To better characterize these discrepancies, the COI values of the FIA antigen test were considered. A proportion of discordant samples (57% for rotavirus and 69% for adenovirus) were also positive for other targets in the GI2 panel (Supplementary Tables S4 and S5). The positivity threshold for both rotavirus and adenovirus antigen tests was defined as a COI ≥ 1 as specified by the manufacturer’s software calculation. Among the 40 discrepant rotavirus samples, the majority (65%) showed low-positive values with a COI between 1 and 5. A smaller fraction (N = 7/40, 17.5%) demonstrated higher COI values (>5), suggesting stronger antigenic signals. Similarly, among the 13 discrepant adenovirus samples, most cases (75%) exhibited COI values in the low-positive range (<5), whereas 25% (N = 3) showed higher COI values (>5).

Specificity values were consistently high across all evaluated targets, ranging from 97.1% to 99.8%, supporting the ability of the GI2 panel to correctly identify negative samples.

## 4. Discussion

The present study provides a comparison between the QIAstat-Dx GI2 panel and conventional diagnostic methods for the diagnosis of acute gastroenteritis. Our findings show that the molecular panel is able to provide a higher pathogen detection rate compared to stool culture and viral antigen assays, and it is also faster in providing results. The overall positivity rate observed with the molecular panel (50.4%) was markedly higher than that obtained with conventional methods (12.3% rotavirus, 4.4% adenovirus and 12.6% stool culture). This difference, of course, is partially due to the broader spectrum of detectable pathogens but also to the higher sensitivity of the real-time PCR approach [6,11,20]. In particular, the possibility to simultaneously detect bacteria, viruses and parasites in a single test represents one of several advantages over traditional diagnostic workflows, which often rely on multiple sequential tests [13,27,28]. For bacterial pathogens such as *Salmonella* spp. and *Campylobacter* spp., a high level of concordance was observed between molecular testing and stool culture, supporting the reliability of the molecular approach for the detection of clinically relevant bacteria. The elevated rate of Campylobacter positivity is consistent with existing literature [29,30]. The GI2 panel does not provide antimicrobial resistance information; therefore, culture-based methods remain necessary when antimicrobial susceptibility testing or epidemiological characterization of bacterial isolates is required, which is essential for patient management and public health surveillance [3,31]. In contrast, agreement between molecular and viral antigen tests was considerably lower. The analysis of discrepant results suggests that this outcome is likely multifactorial; a large proportion of discordant antigen-positive samples were associated with low cut-off index (COI) values, indicating weak signals close to the assay threshold. Furthermore, many of these samples had other pathogen detections with the syndromic panel. This finding could suggest cross-reactivity or aspecific binding effects in antigenic tests contributing to false-positive results, particularly in polymicrobial samples [2,27]. Nevertheless, without confirmation by a third diagnostic method, the true cause of the discordant results remains uncertain. Alternative explanations, including reduced sensitivity of the GI2 panel for specific viral targets, low viral loads, or inhibition of the molecular reaction, cannot be fully excluded. It should also be acknowledged that molecular assays may detect viral nucleic acids in the absence of active infection, reflecting prolonged shedding or low-level carriage [32] therefore, results from both diagnostic approaches require careful clinical interpretation. The high frequency of co-detections observed with the syndromic panel illustrates both the strengths and the challenges of this approach. On one hand, multiplex assays provide a more comprehensive picture of the intestinal microbiological environment, but on the other hand, the clinical relevance of co-infections is not always straightforward. In this context, integration of laboratory findings with clinical presentation remains essential to guide appropriate management decisions [11,32]. Beyond analytical performance, the implementation of syndromic molecular testing has important implications for clinical practice. One of the main advantages of the QIAstat-Dx Gastrointestinal Panel 2 is its rapid turnaround time, enabling the identification of potential pathogens within a few hours. Moreover, the assay provides Ct values for detected targets, which may offer useful complementary information. Although Ct values are not a direct measure of pathogen load and should be interpreted cautiously, they can help contextualize results, particularly in cases of coinfections. In such situations, differences in Ct values may provide an indirect indication of the relative abundance of detected pathogens, potentially supporting clinical interpretation when multiple organisms are identified in the same sample. This can facilitate earlier clinical decision-making, including infection control measures, patient cohorting, and optimization of antimicrobial therapy when indicated. Indeed, rapid pathogen identification may support more targeted therapy and reduce unnecessary empirical antibiotic use, potentially contributing to antimicrobial stewardship efforts. In contrast, conventional methods may require longer processing times or provide more limited diagnostic information. From an economic perspective, molecular syndromic panels are clearly associated with higher direct costs compared to conventional methods, which may limit their widespread use. However, a broader evaluation of cost should consider potential downstream benefits, including reduced need for multiple diagnostic tests, shorter time to diagnosis, improved patient flow and more efficient use of healthcare resources [25]. In this regard, the use of a single comprehensive test may contribute to a more streamlined diagnostic pathway. Nevertheless, formal cost-effectiveness analyses are needed to better define the most appropriate contexts for implementation. Taken together, these considerations suggest that molecular syndromic panels should not necessarily be viewed as a complete replacement for conventional diagnostics, but rather integrated as complementary tools within an integrated diagnostic strategy.

This study has some limitations that should be acknowledged. First, the comparison was performed between the QIAstat-Dx GI2 panel and conventional diagnostic methods without the use of an independent confirmatory assay (e.g., an alternative molecular method or sequencing) to resolve discrepant results. Second, this was a single-center study, and the findings may not be fully generalizable to other healthcare settings or patient populations. In addition, the diagnostic coverage of the two approaches was not identical, as routine conventional testing was limited to bacterial culture and rotavirus/adenovirus antigen detection. Finally, information regarding prior antibiotic exposure and clinical outcomes was not systematically collected, preventing assessment of their potential impact on diagnostic performance and the clinical significance of detected pathogens.

## 5. Conclusions

In conclusion, we believe that the QIAstat-Dx GI2 panel significantly enhances the laboratory diagnosis of gastroenteritis by increasing pathogen detection rates, reducing turnaround time and providing a broad pathogen assessment in a single test. In our opinion, a complementary diagnostic strategy in which syndromic molecular testing is used as a first-line approach for rapid and comprehensive pathogen identification, followed by targeted stool culture in positive cases, is the best strategy to obtain antimicrobial susceptibility data and support timely epidemiological surveillance. Such an integrated workflow leverages the strengths of both methods and may represent a practical and effective approach to optimizing patient management and laboratory resources.

## Figures and Tables

**Table 1:** Results on targets detected by QIAstat-DX GI2 panel, showing the number of positive samples (N), median cycle threshold (Ct), quartile at 25% (Q1) and 75% (Q3) and min–max Ct values. *Entamoeba histolytica*, *Vibrio cholera*, *Vibrio vulnificus* and *Vibrio parahaemolyticus* were not detected and are therefore not included. Ct for *E.coli* O157 was not available. Pos.: positive; n/a: not available.

	Pos. Samples (N)	Median Ct	Q1–Q3	Min–Max
*Clostridium difficile* (tox A/B)	45	25.30	22.90–27.20	19.60–32.00
*Campylobacter* spp.	66	21.85	20.40–25.70	14.60–34.10
*Plesiomonas shigelloides*	2	27.45	27.40–27.50	27.40–27.50
*Yersina enterocolitica*	10	27.80	22.30–37.40	22.30–37.40
Enteroaggregative *E. coli* (EAEC)	56	22.50	18.70–28.30	14.80–31.90
Enteropathogenic *E. coli* (EPEC)	84	26.70	21.75–29.95	14.70–34.50
Enterotoxigenic *E. coli* (ETEC)	19	20.00	17.10–29.90	13.90–32.80
Shiga-toxin producing *E. coli* (STEC) *stx1*/*stx2*	8	24.25	21.70–27.70	21.10–29.20
Enteroinvasive *E. coli* (EIEC)/*Shigella*	14	19.00	16.30–24.30	14.80–25.70
*Salmonella* spp.	19	28.90	26.60–30.60	23.5–31.60
*E. coli* (O157)	2	n/a	n/a	n/a
*Cyclospora cayetanensis*	3	26.90	23.00–30.8	23.00–30.80
*Giardia lamblia*	9	22.10	17.50–24.40	16.00–26.10
*Cryptosporidium* spp.	4	17.90	16.45–19.85	15.60–21.20
Rotavirus A	15	18.60	18.00–32.60	17.10–33.50
Astrovirus	4	14.60	14.30–36.20	14.30–36.20
Norovirus GI/GII	28	20.50	17.30–26.40	12.70–30.50
Adenovirus F40/F41	10	11.85	9.10–17.30	8.50–33.80
Sapovirus (GI. GII. GIV. GV)	17	28.85	21.80–32.25	20.60–35.50

**Table 2:** Performance of the QIAstat-Dx Gastrointestinal Panel 2 as evaluated with prospective clinical specimens collected using FecalSwab or stool samples and traditional methods as the standard. Se: sensitivity, Sp: specificity, PPV: positive predictive value, NPV: negative predictive value.

	Se	Sp	PPV	NPV	*K*-Cohen (95% CI)
*Salmonella* spp.	100%	99.81%	94.74%	100%	0.972 (0.917–100)
*Campylobacter* spp.	100%	97.14%	78.13%	100%	0.863 (0.793–0.933)
Rotavirus	20%	99.00%	71.43%	89.77%	0.273 (0.119–0.427)
Rotavirus (COI > 5, N = 7)	58.8%	98.97%	71.43%	98.20%	0.631(0.429–0.833)
Adenovirus	27.8%	99.7%	83.33%	96.70%	0.403 (0.155–0.651)
Adenovirus (COI > 5, N = 3)	62.5%	99.8%	83.33%	99.25%	0.709 (0.437–0.981)

## Data Availability

The original contributions presented in this study are included in the article/Supplementary Material. Further inquiries can be directed to the corresponding author.

## Supplementary Materials

The following supporting information can be downloaded at https://www.mdpi.com/article/10.3390/microorganisms14061345/s1: Table S1: Sex and age group distribution of the 548 samples included in this study; Figure S1: Monthly distribution of gastrointestinal diagnostic requests. The dashed line represents the geometric mean value of 49.6 samples across the sampling period; Table S2: Number and percentage of detected pathogens with QIAstat-DX GI2 panel; Table S3: Performance of the QIAstat-Dx Gastrointestinal Panel 2 compared to traditional methods set as reference. TP: true positive (positive to both systems), FP: false positive (positive only to GI2), FN: false negative (negative only to GI2), TN: true negative (negatives to both systems), N: number of samples included in analysis; Table S4: GI2 results of the 40 Rota-Ag IFA positive (COI ≥ 1) discordant samples; Table S5: GI2 results of the 13 Adeno-Ag IFA positive (COI ≥ 1) discordant samples.
