# Supplementary material for: QIAstat-Dx Syndromic Molecular Testing Versus Conventional Diagnostics in Acute Gastroenteritis: Impact on Pathogen Detection and Laboratory Workflow
Source: Microorganisms. 2026 Jun 16;14(6):1345. doi: 10.3390/microorganisms14061345 (PMC13305345; doi:10.3390/microorganisms14061345)
Supplement: Supplementary file 1 [file microorganisms-14-01345-s001.zip › microorganisms-4345654-supplementary.pdf]

Male/Female (N)	Percentage %
288/260	52.6/47.4
**Age group (Years)**	Percentage %
0-10	21.7
10-20	18.6
20-30	7.1
30-40	8.7
40-50	7.1
50-60	6.2
60-70	7.8
70-80	10
80-90	9.3
90-100	3.5

Supplementary Table S1: sex and age group distribution of the 548 samples included in this study

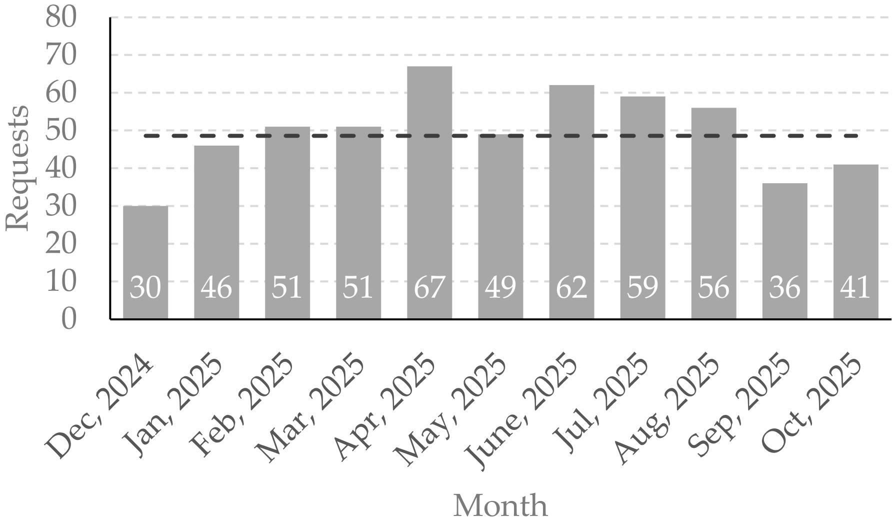

Supplementary Figure S1: monthly distribution of gastrointestinal diagnostic requests. dashed line represent the geometric mean value of 49.6 samples across the sampling period.

Detected/codetected Pathogens (0-6)	Number (N)	Percentage (%)
None	272	49,6
1	171	31,2
2	63	11,5
3	27	4,9
4	12	2,2
5	2	0,4
6	1	0,2

Supplementary Table S2: number and percentage of detected pathogens with QIAstat-DX GI2 panel

	TP	FP	FN	TN	N
***Salmonella* spp.**	18	1	0	520	539
***Campylobacter* spp.**	50	14	0	475	539
**Rotavirus**	10	4	40	351	405
**Rotavirus (COI>5, N=7)**	10	4	7	384	405
**Adenovirus**	5	1	13	386	405
**Adenovirus (COI>5, N=3)**	5	1	3	396	405

Supplementary Table S3: performance of the QIAstat-Dx Gastrointestinal Panel 2 compared to traditional methods set as reference. TP: true positive (positive to both systems), FP: false positive (positive only to GI2), FN: false negative (negative only to GI2), TN: true negative (negatives to both systems), N: number of samples included in analysis.

QIAstat-Dx GI2 target	N
Other Single positive targets	12
Clostridium difficile (tox A/B)	3
EPEC	2
Salmonella spp.	2
Campylobacter spp.	2
Sapovirus	2
All targets Negative	17
TOTAL Rotavirus A negative samples	40

Supplementary Table S4: GI2 results of the 40 Rota-Ag IFA positive (COI≥1) discordant samples

QIAstat-Dx GI2 target	N
Other Single positive targets	4
Clostridium difficile (tox A/B)	3
Campylobacter spp.	2
All targets Negative	4
TOTAL Adenovirus F40/F41 negative samples	13

Supplementary Table S5: GI2 results of the 13 Adeno-Ag IFA positive (COI≥1) discordant samples
